# Association Between Depression and HIV Care Engagement Outcomes Among Patients Newly Initiating ART in Lilongwe, Malawi

**DOI:** 10.1007/s10461-020-03041-7

**Published:** 2020-09-24

**Authors:** Melissa A. Stockton, Bradley N. Gaynes, Mina C. Hosseinipour, Audrey E. Pettifor, Joanna Maselko, Steven M. Mphonda, Kazione Kulisewa, Michael Udedi, Brian W. Pence

**Affiliations:** 1 Epidemiology Department, University of North Carolina at Chapel Hill Gillings School of Global Public Health, 135 Dauer Dr, Chapel Hill, NC 27599 USA; 2 Department of Psychiatry, University of North Carolina at Chapel Hill School of Medicine, 333 S Columbia St, Chapel Hill, NC 27516 USA; 3 University of North Carolina Project-Malawi, Tidziwe Centre, Private Bag A-104, Lilongwe, Malawi; 4 NCDs & Mental Health Unit, Ministry of Health, P. O. Box 30377, Capital City, Lilongwe 3, Malawi; 5 College of Medicine, Department of Mental Health, University of Malawi, P/Bag 360, Chichiri, Blantyre 3, Malawi; 6 Division of Infectious Disease, University of North Carolina at Chapel Hill School of Medicine, 333 S Columbia St, Chapel Hill, NC 27516 USA

**Keywords:** Depression, HIV, Retention, Viral suppression, Sub-saharan africa

## Abstract

As in other sub-Saharan countries, the burden of depression is high among people living with HIV in Malawi. However, the association between depression at ART initiation and two critical outcomes—retention in HIV care and viral suppression—is not well understood. Prior to the launch of an integrated depression treatment program, adult patients were screened for depression at ART initiation at two clinics in Lilongwe, Malawi. We compared retention in HIV care and viral suppression at 6 months between patients with and without depression at ART initiation using tabular comparison and regression models. The prevalence of depression among this population of adults newly initiating ART was 27%. Those with depression had similar HIV care outcomes at 6 months to those without depression. Retention metrics were generally poor for those with and without depression. However, among those completing viral load testing, nearly all achieved viral suppression. Depression at ART initiation was not associated with either retention or viral suppression. Further investigation of the relationship between depression and HIV is needed to understand the ways depression impacts the different aspects of HIV care engagement.

## Introduction

The prevalence of HIV in sub-Saharan countries such as Malawi are among the highest in the world [1, 2]. Like many other countries in the region, Malawi, has adopted a “public health approach” to HIV scale-up in order to meet the UNAIDS 90-90-90 goals (diagnosing 90% of all people living with HIV, providing antiretroviral therapy [ART] to 90% of those diagnosed, and achieving viral suppression for 90% of those treated) [3–5]. Great strides have been made towards achieving these goals and engaging people living with HIV in care across the region [6, 7]; in 2018 in Malawi, 90% of those living with HIV were estimated to be aware of their status, 87% were on treatment and 89% were virally suppressed [1]. Despite recent improvements in ART service provision, both linkage to care and continued engagement in HIV care in sub-Saharan Africa (SSA) remain challenging [8, 9]; improving retention in HIV care will be crucial to attaining the 90% on ART target in the sub-Saharan region [8, 10]. The reasons for attrition among people living with HIV are not entirely understood, though barriers to retention in care may include human resource and institutional challenges, distance to the clinic, lack of support, stigma and fear of HIV status disclosure, and psychiatric illnesses such as depression [11–14].

Depression is a major contributor to the burden of disease and disability and is highly prevalent among people living with HIV in Malawi and elsewhere in SSA, a region where mental health care is often limited [15, 16]. Depression affects 18 to 30% of patients receiving HIV care in Africa [17], and estimates from Malawi range from 1 to 19% [18–21]. The high prevalence of depression among people living with HIV is thought to be due to coping with the HIV diagnosis, disease symptoms, bereavement, relationship crises, stigma and discrimination, co‐existing poverty, ART side effects, fear of death, and infection-related inflammatory processes [22, 23]. Depression can result in reduced quality of life, decreased economic productivity, social isolation, and cognitive decline [22, 23]. Among people with HIV, depression has the potential to worsen HIV-related morbidity and mortality, particularly in low-resource settings.

Depression has been shown to be an important barrier to linkage to care, retention, ART adherence and ultimately long-term viral suppression across the globe [17, 24–29]. A bourgeoning body of research in SSA is beginning to demonstrate that depressed individuals are less likely to be linked to ART care or start ART [30–32]. Further, depression is also associated with poor adherence to ART in SSA [17, 24, 33]. However, limited research has been conducted in SSA on the association between depression and consistent retention in HIV care and viral suppression. As such, further evidence is needed to characterize the association between depression, HIV care engagement, and achievement of viral suppression is the sub-Saharan region.

This analysis addresses this knowledge gap by generating evidence on the association of depression with two key HIV care outcomes: retention in HIV care and viral suppression. Compared to those without depressive symptoms, we hypothesized that those with elevated depressive symptoms at ART initiation would be less likely to be retained in HIV care and to be virally suppressed 6 months after ART initiation.

## Methods

### Study Design

**Fig. 1:**
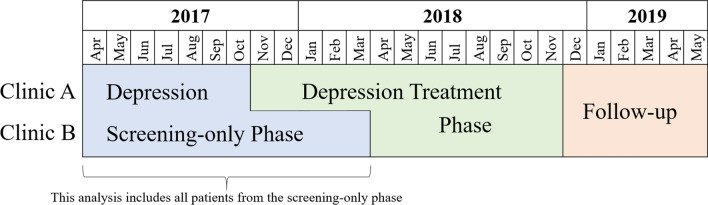
Program implementation

This study is nested within the first phase of a pilot program that integrated depression screening and treatment into routine HIV primary care using existing staff at two public health clinics in Lilongwe, Malawi. We implemented this program in two staggered phases—a depression screening-only phase and a depression treatment intervention phase [34]. This study focused on individuals who initiated ART during the screening-only phase (Fig. 1).

**Fig. 2:**
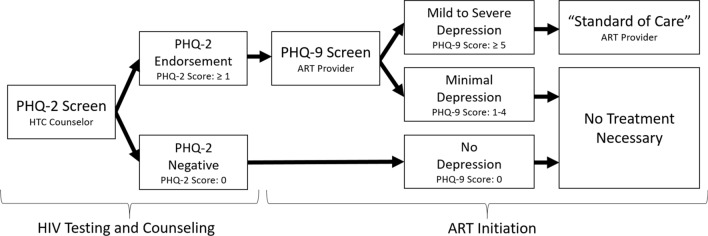
Depression screening process

During the screening-only phase, HIV testing and counseling (HTC) counselors and ART providers screened patients for depression at ART initiation using the Patient Health Questionnaire-9 (PHQ-9). The PHQ-9 is a nine-item questionnaire that assesses the presence and frequency of the nine Diagnostic and Statistical Manual of Mental Disorders symptoms of major depression [35]. It has been widely used in the sub-Saharan region [36–38]. HTC counselors screened all patients newly diagnosed with HIV for depressed mood or anhedonia using the first two questions of the PHQ-9 known as the Patient Health Questionnaire-2 (PHQ-2). For patients who endorsed at least one of the PHQ-2 questions, the ART provider then administered the remaining seven PHQ-9 questions during ART initiation. A total score of 5–9 and ≥ 10 are considered indicative of mild depression and moderate to severe depression, respectively [39]. Patients who were identified with elevated depressive symptoms were managed by providers using existing “standard of care” options within the Malawi primary care system. (Figure 2) These options theoretically included counseling, antidepressants or referral to an on-site or off-site mental health specialist, or in acute cases, transport to the outpatient psychiatric unit at the nearby district hospital. However, in practice counseling consisted of informal counseling by the ART provider and antidepressants were rarely prescribed (and when prescribed, often at sub-therapeutic doses). In theory, the ART providers could have used the treatment program’s evidence-based protocol to prescribe antidepressants or refer patients for evidence-based counseling at follow-up appointments during the treatment phase, although we identified only a few instances when this occurred.

### Population

All non-pregnant adults (18 years or older) newly initiating ART at the study sites were eligible to be enrolled in the program evaluation. Research assistants approached such individuals awaiting ART initiation to invite them to participate in the study and nearly all (96%) provided informed consent. This analysis is restricted to consenting individuals who completed depression screening during the screening-only phase of the program, e.g. only those who initiated care between April 2017 and October 2017 at Clinic A and between April 2017 and March 2018 at Clinic B (Fig. 1).

### Data Collection and Abstraction

This study relied on the abstraction of routinely collected clinical data on depression screening and HIV care from consenting participants’ ART clinical records over a 13-month period, starting at ART initiation. At the study sites, when a patient initiates ART, clinic staff create a paper medical chart (called an ART mastercard) and accompanying electronic medical record for the patient where HIV care data will be recorded. At each appointment, the clinicians schedule the next follow-up appointment and provide the patient with a supply of ART that will last until their next appointment. ART pills are provided in increments of 30. Generally, for the first 6 months of care, newly initiating ART patients are scheduled to come monthly, though exceptions can be made and a larger supply of ART can be provided.

### Measures

#### Depression at ART Initiation

Elevated depressive symptoms suggesting a high risk of a depressive disorder (hitherto described as “depression”) was defined as a PHQ-9 score ≥ 5 at ART initiation, following the definition in the parent study [40]. We also consider a 3-level categorical depressive severity measure (0–4, 5–9, and ≥ 10, corresponding to no, mild, and moderate to severe depression, respectively).

#### Viral Suppression

We defined viral suppression as a viral load < 1000 copies/mL drawn at least 5 and a half months (166 days) after starting ART, following the 2018 Malawian ART guidelines. Viral load testing was performed at Bwaila Hospital in Lilongwe using collected plasma or dried blood spots and processed by Abbott m2000 RealTime HIV-1 assay instruments [41].

#### Retention in HIV Care

We constructed several measures to capture retention in HIV care through 6 months in care. Some measures were constructed specifically for this analysis, while others were constructed in alignment with nationally and internationally recognized measures of retention.

Continuous engagement in care was defined as never being more that 14 days late to an appointment through 6 months in care.

“Alive and in care” at 6 months was defined as being on ART for at least some portion of the 2 months prior to the 6-month anniversary of starting ART, in alignment with Malawi Ministry of Health reporting practices. According to the Malawi Guidelines for Clinical Management of HIV in Children and Adults, an ART patient is classified as a “defaulter” if they go 2 months without ART based on the pills provided at their most recent appointment [42]. Patients who are not known to have transferred, stopped ART, or died are classified as “alive and on ART.”

Currently on ART at 6 months was defined as currently having ART on hand at 6 months, i.e. attending an appointment prior to the 6-month point and receiving a supply of ART that would last through the 6-month point. For example, if a participant attended an appointment 5 and half months after starting ART and received a 30-day supply of ART, they would have been considered “currently on ART” at 6 months. This indicator aligns closely with the PEPFAR definition of “currently on treatment” [43].

In care after 6 months was defined as attending at least one appointment where ART is provided after 6 months in care. This definition aligns more closely with the UNAIDS Global AIDS Monitoring 2019 Indicators by identifying participants known to be on ART at some point 6 months after initiation [44].

HIV appointment attendance was defined as the proportion of scheduled HIV appointments attended within one week of the scheduled appointment date in the first 6 months of care [45].

Consistent ART was defined as never going more than 5 days without ART in the first 6 months, calculated from the cumulative days’ supply of ART dispensed at each appointment in the first 6 months and the time between appointments.

ART pill possession ratio was defined as the proportion of the first 6 months (183 days) since ART initiation with ART in hand, calculated from cumulative pills dispensed at each ART appointment through 6 months [46].

#### Retention + Viral Suppression

A combined outcome of continuous engagement in HIV care with viral suppression 6 months after starting ART was defined as meeting both of the following criteria: never being more than 14 days late to a scheduled HIV appointment through 6 months in care and having a viral load < 1000 copies/mL drawn at least 5.5 months (166 days) after ART initiation.

#### Variables of Interest

Due to the data collection design of this study, only routinely collected clinical data was captured including age, sex, clinic, World Health Organization (WHO) HIV clinical stages for HIV surveillance [47], village of residence, and ART medication prescribed. Village of residence was used to categorize individuals as residing in urban Lilongwe City, in rural Lilongwe District or outside of Lilongwe District. Baseline CD4 and viral load are not collected in the Malawi public health system as the policy encourages a ‘test and treat’ strategy; individuals who test positive for HIV are initiated on ART irrespective of CD4 and viral load.

### Analysis

We first completed unadjusted, tabular comparisons of HIV care outcomes by depressive severity (none, mild, and moderate to severe). We then used log-binomial models to estimate adjusted risk ratios comparing the probability of each binary HIV care outcome among those with depression compared to those without. For the continuous outcomes, we used ordinary least-squares linear regression models to estimate mean differences. Potential confounders were identified through directed acyclic graph (DAG) analysis.

To address missing outcome data for those who transferred to a non-study facility or who returned for care around 6 months but did not get a viral load, we used multiple imputation by chained equations (MICE) to fill missing values using logistic regression imputation methods [48]. Assuming this data was missing at random (MAR), we imputed values based on the covariates included in the final model. We generated 15 imputed datasets to ensure the number of imputed datasets was at least as large as the percentage of incomplete information for the main outcome, “continuous engagement with viral suppression”[49]. Finally, we confirmed that the number of imputed datasets was also larger than the parameter-specific fraction of missing information for all parameters included in the final model [50].

All analyses were performed using STATA IC 14.

### Ethical Review

The National Health Sciences Research Committee of Malawi (NHSRC) and the Biomedical Institutional Review Board (IRB) of the University of North Carolina at Chapel Hill approved the study protocol. All participants underwent a consent process during ART initiation and gave written informed consent to allow the abstraction of their clinical data. During the consent process, the research assistants explained that participation was voluntary, would not affect care provided at the facility, and would not be necessary to receive ART.

## Results

### Participant Characteristics

Of 1091 participants screened, 602 (55%) screened negative on the PHQ-2; the full PHQ-9 was administered to the remaining 489. (Table 1) Of these, 290 (27% of those enrolled) endorsed symptoms consistent with our operational definition of depression (PHQ-9 score ≥ 5). Of these, 74% had mild depression (PHQ-9 scores 5–9) and 26% had moderate to severe depression (PHQ-9 scores ≥ 10). The most commonly recorded depression treatment was informal “counseling by the ART provider.” However, a total of 16 individuals with depression were prescribed antidepressants, 13 of which started a sub-therapeutic dose. Only two of these individuals were prescribed antidepressants more than once. None of the participants were ever referred for evidence-based counseling and only six individuals were ever prescribed antidepressants at a follow-up visit. Just over half of all participants were female; those with depression were slightly more likely to be female than those without. The mean age of participants was 33.5 years and did not vary appreciably between participants with and without depression. Nearly all participants were classified as asymptomatic (Stage I) for HIV at ART initiation and were initiated on a combination of tenofovir, lamivudine and efavirenz (TDF/3TC/EFV).

**Table 1:** Participant characteristics (N = 1091)

n(%) or mean (sd)	Overall (N = 1091)	Not depressed (N = 801)	Depressed (N = 290)
Baseline depression severity			
Screen-negative (PHQ-2: 0)	602 (55%)	602 (75%)	N/A
Minimal (PHQ-9: 1-4)	199 (18%)	199 (25%)	N/A
Mild (PHQ-9: 5-9)	214 (20%)	N/A	214 (74%)
Moderate (PHQ-9: 10-19)	71 (7%)	N/A	71 (24%)
Severe (PHQ-9: 20-27)	5 (< 1%)	N/A	5 (2%)
Baseline depression treatment			
Counseling by ART provider	251 (23%)	N/A	251 (87%)
Antidepressants	16 (1%)	N/A	16 (6%)
None	824 (76%)	801 (100%)	23 (8%)
Sex			
Male	513 (47%)	388 (48%)	125 (43%)
Female	578 (53%)	413 (52%)	165 (57%)
Age	33.5 (9.6)	33.4 (9.4)	33.6 (10.0)
Residence*			
Urban lilongwe	976 (93%)	717 (93%)	259 (93%)
Rural lilongwe	54 (5%)	40 (5%)	17 (6%)
Outside lilongwe district	17 (2%)	15 (2%)	2 (1%)
WHO disease stage			
I	1089 (>99%)	801 (100%)	288 (99%)
II–IV	2 (< 1%)	0 (0%)	2 (1%)
Baseline ART prescription**			
TDF/3TC/EFV	1078 (99%)	791 (99%)	287 (99%)
Other	12 (1%)	9 (1%)	3 (1%)
Clinic			
Clinic A	435 (40%)	319 (40%)	116 (40%)
Clinic B	656 (60%)	482 (60%)	174 (60%)

*Missing n = 44

**Missing n = 1

### HIV Care Outcomes: Retention in Care and Viral Suppression

The HIV care outcomes measures did not vary appreciably between those without, with mild, and with moderate to severe depression (Table 2). Around 7% of participants transferred (n = 72) or died (n = 2) within the first 6 months of care and thus had missing outcome data for every metric. Regardless of metric, retention in HIV care was generally low. Using the Malawi Ministry of Health ART patient classification, around 60% of participants would have been classified as “alive and in care” at 6 months and attended an appointment after 6 months. Around 50% were currently on ART at 6 months and around 40% maintained a consistent supply of ART through 6 months. There was also substantial missing viral load data as only 45% (N = 454) of patients who had not transferred or died had viral loads drawn. Of those with viral load data, nearly all were virally suppressed. Only around 21% of participants achieved both continuous engagement and viral suppression, though an additional 7% (n = 72) achieved continuous engagement, but had missing viral load data.

**Table 2:** HIV care outcomes, by depressive severity

Outcome	Total	Depression		
		None	Mild	Moderate to Severe
		% or Mean (SD)		
Retention indicators	N = 1017	N = 751	N = 199	N = 67
Continuous engagement	30%	28%	34%	34%
“Alive and in care”	59%	57%	63%	58%
Currently on ART	48%	46%	53%	48%
In care after 6 months	57%	57%	57%	61%
Consistent ART	40%	40%	43%	40%
HIV appointment attendance (Range: 0-1)	0.5 (0.4)	0.5 (0.4)	0.6 (0.4)	0.5 (0.4)
ART pill possession (Range: 0.16-1),	0.7 (0.4)	0.7 (0.4)	0.7 (0.4)	0.7 (0.4)
Viral suppression	N = 454	N = 339	N = 88	N = 27
Viral suppression	93%	93%	94%	93%
Retention + Viral suppression	N = 1017	N = 751	N = 199	N = 67
Continuous engagement with viral suppression	21%	20%	26%	24%
Continuous engagement, missing viral load	7%	7%	7%	9%

Transferred within the first 6 months of care: Not depressed n = 48; Mild Depression n = 15; Moderate to Severe Depression n = 9; Died within the first 6 months of care: Not depressed n = 2; Denominators vary due to viral loads not being drawn or not having or attending a scheduled appointment around 6 months

The final adjusted model controlled for clinic and sex. As a continuous variable age introduced model instability and was removed from the final adjustment set as age did not appear to be associated with any of the outcomes or depression at baseline. WHO stage, ART medication, and area of residence did not vary enough to be considered potential confounders. Addressing missing data through multiple imputation did not appreciably change any of the results.

After adjustment, all the estimates for the relationship between mild to severe depression at ART initiation and HIV care outcomes were both close to the null and had 95% confidence intervals that spanned the null. (Table 3) Sensitivity analyses treating depression as a categorical variable (no, mild, and moderate to severe depression) and as a binary variable comparing moderate to severe depression (PHQ-9 ≥ 10) to no to mild depression (PHQ-9 < 9) yielded similar results.

**Table 3:** Association of depression (PHQ-9≥5) at ART initiation with HIV care outcomes

Outcome	Unadjusted	Adjusted*	Imputation**
	RR (95% CI)		
Retention indicators			
Continuous retention	1.22 (1.00–1.50)	1.16 (0.96–1.41)	1.17 (0.96–1.41)
“Alive and in care”	1.08 (0.97–1.21)	1.08 (0.97–1.21)	1.09 (0.98–1.21)
Currently on ART	1.12 (0.97–1.29)	1.11 (0.97–1.27)	1.12 (0.98–1.28)
In care after 6 months	1.02 (0.91–1.15)	1.04 (0.92–1.17)	1.04 (0.93–1.17)
Consistent ART	1.06 (0.90–1.25)	1.05 (0.89–1.23)	1.05 (0.89–1.23)
Retention + Viral Suppression			
Continuous retention with viral suppression	1.29 (1.01–1.65)	1.21 (0.96–1.54)	1.19 (0.97–1.45)
Mean difference (95% CI)			
HIV appointment attendance (Range: 0–1)	0.03 (− 0.03–0.08)	0.03 (− 0.03–0.08)	0.06 (− 0.02–0.09)
ART pill possession (Range: 0.16–1)	0.01 (− 0.04–0.06)	0.01 (− 0.04–0.06)	0.02 (− 0.03–0.06)

*Adjusted for clinic and sex

**Further corrected for missing data due to transfer or missing viral load via multiple imputation

## Discussion

While the prevalence of depression among this population of adults newly initiating ART was high at 27%, those with depression had similar HIV care outcomes at 6 months compared to those without depression. Retention metrics were generally poor for both groups. However, among those sent for viral load testing, nearly all achieved viral suppression.

The influence of depression on retention in HIV care is complicated. Several recent reviews and meta-analyses have documented the association between depression and ART adherence [24, 51], though the relationship between depression and engagement in HIV care or retention is less well understood. We hypothesized that depression could plausibly impair adherence and appointment attendance as depression often manifests through loss of interest, poor concentration, poor motivation, reduced self-efficacy, fatigue, hopelessness, and suicidality [24, 26, 52]. However, in our study population depression was not associated with any of the retention indicators or viral suppression at 6 months. Several studies conducted in South Africa, Kenya, and Uganda examining the association between depression prior to HIV testing and linkage to care had mixed results [32, 53, 54]. In South Africa, where linkage to care was defined as obtaining a CD4 count within 3 months of a positive HIV test, one study found no difference between those with and without depression at testing [53] and one found that those with depression were less likely to be linked to care [32]. In Kenya and Uganda, greater depressive symptom severity was associated with greater likelihood of ART initiation during the study period among sero-converted partners of previously sero-discordant couples [54]. Studies of depression and retention in care conducted in Malawi and the Democratic Republic of the Congo found no difference between 12-month retention or viral suppression among pregnant women with and without depression at ART initiation [20, 55]. One explanation for the differences in the literature relating depression and ART adherence versus relating depression and retention is that depression may not affect engagement in care in the same way it impacts ART adherence; different skills sets are required for adherence to daily ART than maintaining monthly clinic visits [53]. Further efforts to understand the relationship between depression and HIV will need to unpack the mechanisms through which depression impacts the different aspects of HIV care engagement.

Nonetheless, the lack of association between depression at ART initiation and any of the HIV outcomes in this study population is striking. Qualitative interviews with providers and patients conducted within the larger parent study during the screening phase of the program, suggest that individuals identified with depression during the screening phase possibly received additional counseling on accepting their HIV status, ART adherence, and managing their depression [56]. While these participants with depression did not receive evidence-based standardized depression treatment, it is possible that this additional attention may have acted as an informal intervention. As such, this additional attention may have had a positive impact on HIV care engagement for depressed patients included in this analysis relative to those without depression.

Locally adapted, valid depression diagnostic and management tools are vital for addressing the burden of depression among people living with HIV. The implementation team chose the PHQ-9 for use among people living with HIV because it focuses specifically on depression, has been widely used and validated in many different cultures (including among people living with HIV in SSA) [36, 37, 57], and works well both as a case identification tool as well as a longitudinal monitor of response to treatment. However, at the time, the PHQ-9 had yet to be validated in Malawi, though it has since undergone validation among a population of patients with diabetes [58]. Along that vein, cases of “depression” were identified with the PHQ-9 screening tool using a cutoff score of 5 and not a diagnostic interview. It is plausible that some depressive symptoms were not actually features of a major depressive episode, but rather a manifestation of milder syndromes, more likely to resolve spontaneously and not require an intervention. However, the sensitivity analyses using a cutoff score of 10 as well as the recent validation study support our use and interpretation of the tool. Furthermore, the PHQ-9 was originally developed to be self-reported, but was administered by providers due to low levels of literacy among the patient population. Given the overlap in symptomology between HIV and depression itself, HIV providers may have identified an inaccurate burden of depression among people living with HIV [59]. As this study relied on existing staff to screen patients for depression who were not incentivized to engage in the depression screening program, it is also possible that providers underdiagnosed cases of depression. While a small sub-study comparing the providers’ administration of the PHQ-9 to that of trained research assistants did find that research assistants identified more cases of depression than providers, there was still high overall agreement between providers and research assistants [60]. Nevertheless, the accuracy of the PHQ-9 may have been compromised, given the mode of administration by existing staff (as opposed to self-reported) with varying degrees of commitment to the program. Tools such as the PHQ-9 would benefit from further quantitative validation against a gold standard diagnostic instrument to confirm their utility as part of task-shifting programs in SSA, particularly among people living with HIV.

Finally, complexities in measuring engagement and retention in HIV further complicate the study of the role of depression in HIV care. Despite the importance of retention to successfully treating HIV, there is no recognized “gold standard” measure for retention in care [45]. A recent meta-analysis highlighted some of the challenges around comparing studies examining the association between mental health disorder diagnoses and retention in HIV care, noting that “retention in care” may be operationalized to include measures of visit constancy, kept visits, no-show rates, and gaps in care [29]. In this study, retention overall was very low; only 30% of all participants remained continuously engaged in care through 6 months and only 43% attended an appointment after 6 months in care. Using Malawi’s definition of retention, overall 58% of participants were considered alive and in care at 6 months, though this is still lower than Malawi’s 2018 12-month retention estimates which found that 72% of adults who initiated ART were still in care at 12 months [61]. It is possible that we potentially underestimated retention in care due to “silent transfers,” or individuals who decided to access care at a different location without formally transferring their records. In fact, the Malawi Ministry of Health assumes that actual retention is about 10% higher due to this misclassification of ‘silent transfers’ as ‘defaulters’ in clinic-based retention analysis, though a meta-analysis of low- and middle-income country studies suggests retention may be as much as 18% higher [62]. Recognizing that this misclassification could have influenced all of the HIV care engagement measures, the rate of silent transfer should not have varied between groups, so “silent transfers” should not have significantly biased comparisons between those with and without depression [61]. In this sense, “retention” in our study is tantamount to “retention at the specific clinic,” and the data available may not yield a complete picture of engagement in care, limitations often faced by studies on retention in HIV care in SSA [63, 64]. Innovative methods and approaches for measuring retention in care to manage these complexities are needed, particularly in low resource settings.

### Limitations

Due to the implementation science nature of this study, the covariates captured were limited to routinely collected clinical data. It is possible that the presented analyses were biased by unmeasured confounding factors such as stigma, socio-economic status, or transportation barriers. It is also possible that we overestimated viral suppression as viral loads could only be drawn from patients who remained in care and a large proportion of patients who did return for care were never sent for viral load testing.

## Conclusion

This study documented a high prevalence of depression among patients newly initiating ART and low retention in care at 6 months. However, the examined HIV care outcomes at 6 month were similar between those with and without depression. Further research is needed to understand the mechanisms through which depression may undermine different aspects of the HIV care continuum, from testing through sustained retention and ultimately viral suppression.
